# Walnut Oligopeptide Delayed Improved Aging-Related Learning and Memory Impairment in SAMP8 Mice

**DOI:** 10.3390/nu14235059

**Published:** 2022-11-28

**Authors:** Qian Du, Meihong Xu, Lan Wu, Rui Fan, Yuntao Hao, Xinran Liu, Ruixue Mao, Rui Liu, Yong Li

**Affiliations:** 1 Department of Nutrition and Food Hygiene, School of Public Health, Peking University, Beijing 100191, China; 2 Beijing Key Laboratory of Toxicological Research and Risk Assessment for Food Safety, Peking University, Beijing 100191, China

**Keywords:** walnut oligopeptide, SAMP8, mitochondrial function, learning and memory

## Abstract

Aging-related learning and memory decline are hallmarks of aging and pose a significant health burden. The effects of walnut oligopeptides (WOPs) on learning and memory were evaluated in this study. Sixty SAMP8 mice were randomly divided into four groups (15 mice/group), including one SAMP8 age-control group and three WOP-treated groups. SAMR1 mice (*n* = 15) that show a normal senescence rate were used as controls. The SAMP8 and SAMR1 controls were administered ordinary sterilized water, while the WOP-intervention groups were administered 110, 220, and 440 mg/kg·bw of WOPs in water, respectively. The whole intervention period was six months. The remaining 15 SAMP8 (4-month-old) mice were used as the young control group. The results showed that WOPs significantly improved the decline in aging-related learning/memory ability. WOPs significantly increased the expression of BDNF and PSD95 and decreased the level of APP and Aβ1-42 in the brain. The mechanism of action may be related to an increase in the activity of antioxidant enzymes (SOD and GSH-Px), a reduction in the expression of inflammatory factors (TNF-α and IL-1β) in the brain and a reduction in oxidative stress injury (MDA). Furthermore, the expression of AMPK, SIRT-1, and PGC-1α was upregulated and the mitochondrial DNA content was increased in brain. These results indicated that WOPs improved aging-related learning and memory impairment. WOP supplementation may be a potential and effective method for the elderly.

## 1. Introduction

Aging is a major risk factor for gradual degenerative changes in the nervous system. The number of elderly individuals with neurodegenerative disorders, such as mild cognitive impairment and Alzheimer’s disease, is increasing, thus causing a huge economic burden worldwide [1]. Neural dysfunction or degeneration manifests as mitochondrial dysfunction [2], accumulation of oxidatively damaged molecules [3], dysregulated energy metabolism [4], autophagy dysfunction [5], impaired DNA repair [6], disruption of neuronal networks [7], altered calcium homeostasis [8], and inflammation [9]. The nervous system is a highly energy-demanding system, and brain tissue is sensitive to oxidative stress. As aging occurs, excessive and non-self-cleared free radicals accumulate in the brain, thus triggering a vicious cycle, which perpetuates neuron damage, dysfunction of the nervous system, and a decline in learning and memory ability. Therefore, exploring the function of antioxidants with high safety with the aim to slow down oxidative stress in the brain is a good nutrition-based strategy to improve age-related learning and memory impairment. Based on high safety, dietary pattern [10], or inclusion of food-active substances, such as vitamin E [11], coenzyme Q10 [12], curcumin, and ginsenosides [13,14], nutritional intervention is an optimal choice for the prevention and treatment of neurodegenerative diseases.

As optimal food ingredients, bioactive peptides have been widely used based on their various characteristics, such as high absorptivity, hypo-allergenicity, and diverse biological activity [15]. Walnut (Juglans regia L) oligopeptides (WOPs) are enzymatically hydrolyzed from walnut seed residues after oil extraction. Our previous studies have suggested that WOPs exhibit practical beneficial functions, such as anti-hypoxia, anti-fatigue, regulation of blood lipids, promotion of probiotic proliferation, and a reduction in systematic inflammatory and metabolic disorders, and other biological activities, in addition to im-proving the nutritional profile [16,17,18,19,20]. WOPs have shown excellent potential antioxidative ability and maintain mitochondrial function.

SAMP8 mice are used as a rapidly aging mouse model that has the advantage of short lifespan and accelerated aging process. It is an excellent model to study memory deficits and a suitable animal model for mild cognitive impairment (MCI) associated research [21]. Additionally, mitochondrial dysfunction is the main cause of high oxidative stress in SAMP8 mice and is related to aging and learning/memory impairment [22,23]. Therefore, the present study aimed to investigate the impact of WOPs on age-related learning and memory impairment and to explore the possible underlying mechanisms involving mitochondrial targets in SAMP8 mice.

## 2. Materials and Methods

### 2.1. Materials

WOPs were provided by Beijing Huataitai Biotechnology Co., LTD (Beijing, China), in a faint yellow solid powder, and mainly consist of macromolecule peptides with molecules below 1000 D. WOPs are rich in glutamic acid, aspartic acid, arginine, and leucine. The amino acid composition of WOPs has already been described in our pervious study [16,17,18,19,20].

Assay kits used to determine oxidative stress and cholinergic were purchased from Nanjing Jiancheng Biological Engineering Research Institute (Nanjing, China), including superoxide dismutase (SOD), glutathione peroxidase (GSH-Px), malondialdehyde (MDA), acetylcholinesterase (AChE), and choline acetyl-transferase (ChAT). The proinflammatory factors assay, tumor necrosis factor α (TNF-α) and interleukin (IL)-1β were purchased from MultiSciences (Lianke) Biotech Co., Ltd. (Hangzhou, China) and Beijing Zhongshang Boao Bio Technology Co., Ltd. (Beijing, China), respectively. TRIzol and the DNeasy Tissue Kit were purchased from Invitrogen (Carlsbad, CA, USA) and QIAGEN Sciences (Germantown, MD, USA), respectively. The secondary antibody (anti-rabbit or anti-mouse) was obtained from CST, including brain-derived neurotrophic factor (BDNF), postsynaptic density-95 (PSD95), APP and Aβ1-42.

### 2.2. Animal

A total of 60 four-month-old male SAMP8 and fifteen SAMR1 mice were provided by the Medical Laboratory Animal Science Department of Peking University. After one-week adaptive feeding, SAMP8 mice were randomly equally divided into one SAMP8 control and three WOP groups, according to body weight. All the mice were housed at constant temperature (25 ± 1 °C) and humidity (50–60%) under a 12 h:12 h light-dark cycle, and allowed free access to standard food (AIN-93G diet) throughout the experiment.

SAMP8 and SAMR1 controls were administered ordinary sterilized water, while WOP-intervention groups were administered 110, 220, and 440 mg/kg·bw concentrations of WOPs in water, respectively). The food consumption of water and food, as well as body weight were recorded weekly. Another fifteen 4-month-old SAMP8 mice were used as the young control group. After six months of continuous intervention, CO_2_ was used to anesthetize the mice. Anesthetized mice were euthanized by cervical dislocation after blood sampling. The hippocampus was isolated and immediately placed on ice, before storing at −80 °C.

### 2.3. Behavioral Tests

After six months of continuous intervention, four behavioral tests were carried out for testing the ability of learning and memory, including the open-field test, the Morris water maze test, the shuttle box test, and the step-down test. The specific process of the experimental operation was described previously [24]. To reduce the systematic error and ensure consistency of the observations before and after the intervention, all tests were performed by the same personnel. During the whole process of investigation, the movement of personnel was reduced and silence was maintained in the test room. We applied a 2-day interval between behavioral tests to eliminate any interference.

### 2.4. Biochemical Assays and Enzyme-Linked Immunosorbent Assay

All indicators, including the levels of oxidative stress biomarkers (SOD, GSH-Px, and MDA) in the serum and brain, and the levels of inflammatory parameters (TNF-α and IL-1β), and cholinergic system-related enzymes (AChE, ChAT) in the brain, were deter-mined using assay kits, according to the protocol provided by the manufacturer.

### 2.5. Western Blot Analysis

Western blot analysis was performed for three mice in each group, which were randomly selected. After extracting and measuring, the total proteins were transferred to PVDF membranes through gel electrophoresis. After being blocked with TBST, the membranes were incubated with primary antibodies overnight at 4 °C. Then, the membranes were incubated with secondary antibodies for 4 h at 4 °C. Routinely, protein load was detected by using enhanced chemiluminescence (ECL) detection.

### 2.6. Quantitative Real-Time PCR and Analyses of mtDNA Content

Total RNA and DNA were isolated from the hippocampal tissue of SAMP8 mice. The expression of target genes and mtDNA copy number were detect by real-time reverse transcription-PCR, according to our previous study [20]. Cycling conditions were 95 °C for 5 min, followed by 40 cycles of 95 °C for 10 s and 60 °C for 30 s. mRNA expression levels were quantified using a real-time PCR amplification kit with an ABI 7500 Real-Time PCR System (Applied Biosystems, Carlsbad, CA, USA).

The specific primers were used as follows: AMPK forward 5′-GAAAGTGAAGGTGGGCAAGC-3′ and reverse 5′-GATGTGAGGGTGCCTGAACA-3′; PGC-1α forward 5′-TCACGTTCAAGGTCACCCTA-3′ and reverse 5′-TCTCTCTCTGTTTGGCCCTT-3′; SIRT1 forward 5′-AGCGTCTTGACGGTAATCAA-3′ and reverse 5′-AACTTGGACTCTGGCATGTG-3′; mtDNA forward 5′-CGTTAGGTCAAGGTGTAGCC-3′ and reverse 5′-CCAGA CACACTTTCCAGTATG-3′; GAPDH forward 5′-TGCCCCCATGTTTGTGATG-3′ and reverse 5′-TGTGGTCATGAGCCCTTCC-3′.

### 2.7. Statistical Methods

Experimental results were expressed as the mean and standard deviation. SPSS software version 24.0 (SPSS Inc., Chicago, IL, USA) was used for analysis. Data with homogeneity of variance were analyzed by one-way ANOVA; otherwise, data were analyzed using a non-parametric test. LSD was used for comparison between the two groups, and statistical significance was set at *p* < 0.05.

## 3. Results

### 3.1. Effects of WOPs on the Body Weight, Food and Water Consumption of Mice

There was no significance in body weight, food, and water consumption changes among the control groups and three WOP intervention groups (data not shown).

### 3.2. Effects of WOPs on Learning and Memory Performance

#### 3.2.1. The Space Exploration Capability of the Open-Field Test

As shown in Table 1, even though the number of squares crossed and rearing were present a downtrend in the SAMP8 group in the comparison with the young and SAMR1 control groups without remarkable differences (*p* > 0.05), they were significantly lower in WOPs-HG than that in young control (*p* < 0.05). Furthermore, the number of squares crossed in all WOP groups were markedly lower than those in SAMR1 control (*p* < 0.05). There were no differences among the groups in the number of fecal boli and inner squares entered, as well as the times spent in the inner squares (*p* > 0.05).

#### 3.2.2. The Learning and Memory Capability in the Morris Water Maze Test

**Figure 1:**
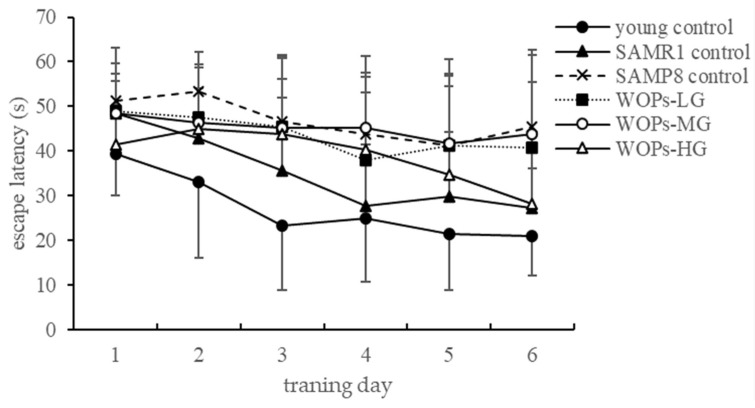
The trend of escape latency. Data are presented as the means ± SD (*n* = 12).

During the place navigation test, there is no significant difference in swimming speed between the groups. It can be considered that swimming speed will not interfere with the time for mice to find the platform, that is, the result of the escape latency. Thus, the escape latency of the mice can be used to reflect the spatial learning and memory level of the mice. With the increase in training days, the duration of escape latency in each group showed a decreasing trend (Figure 1). During the spatial probe test, the escape latency of SAMP8 old mice was significantly higher than that of the young and SAMR1 control groups. Among them, the escape latency of mice in WOP-treated groups was shortened in the comparison with SAMP8 control. The escape latency of the WOPs-HG group was significantly different from that of the SAMP8 control group (*p* < 0.05).

The space exploration experiment is used to measure the memory retention ability of the platform space position of the mice. After the platform was removed on the 7th day of the experiment, the target quadrant residence time and the number of crossings of the SAMP8 elderly control group were lower than those of the young control group, while the WOPs-HG group SAMP8 mice crossed. The number of platform locations increased compared with the elderly control group. The three intervention groups all made the SAMP8 mice stay longer in the target quadrant, indicating that WOP intervention in SAMP8 elderly mice can improve the learning and spatial memory decline of mice, but the intervention effect has no obvious dose–response relationship (Table 2).

#### 3.2.3. The Trend of AAR and PAR in the Shuttle Box Test

**Figure 2:**
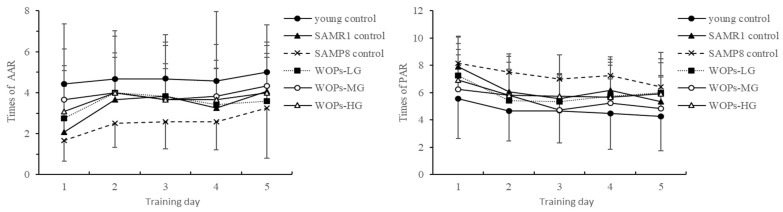
The trend of AAR and PAR. Data are presented as the means ± SD (*n* = 12).

The number of active avoidances of mice in each group gradually increases with the number of days, and the number of electric shocks gradually increases with the number of days (Figure 2). A decrease indicates that the learning effect of the mice on the acousto-optic conditioned reflex is continuously enhanced; compared with the young group, the number of active avoidances of the old control SAMP8 mice is reduced, and the number of electric shocks is increased. WOPs-MG and the intervention of WOPs-HG increased the number of active avoidances of SAMP8 mice and reduced the number of electric shocks, which can reach the level of SAMR1 and the young control group, suggesting that WOPs can enhance the weakened escape of electrical stimulation in old SAMP8 mice, showing its ability to improve conditioned reflexes.

#### 3.2.4. The Number of Errors and Latency in the Step-Down Test

A platform-jumping experiment was conducted to test the passive avoidance reaction ability of mice in each group. The test at 24 h after training found that compared with the youth group, there was no statistical difference in the number of mistakes in the SAMR1 control group. However, the number of errors of the elderly SAMP8 mice has increased significantly, and the latency of staying on the platform is relatively short. WOPs-MG and WOPs-HG reduced the number of times that elderly mice received electric shocks, and the residence latency of elderly mice in the three WOP intervention groups was extended, making it reach the level of SAMR1. This indicates that WOPs can improve the reduction in passive avoidance response in SAMP8 mice (Table 3).

### 3.3. Effects of WOPs on Oxidative Stress in Serum and Hippocampus Tissue

Compared with young control, the SOD activity of SAMP8 control significantly decreased in serum (*p* = 0.001 vs. young control and *p* = 0.000 vs. SAMR1 control). Moreover, SOD activity was particularly higher in WOP groups than SAMP8 control in serum (*p* < 0.05). Similarly, there were significant differences between the SAMP8 control group and the young control on GSH-Px activity in the serum (*p* = 0.001 vs. young control and *p* = 0.001 vs. SAMR1 control). Compared with the model SAMP8 control group, the GSH-Px activity of serum significantly, respectively, increased in the WOPs-MG and WOPs-HG groups (*p* < 0.05). There was similar tendency on the SOD and GSH-Px activity in hippocampus tissue. However, due to the high standard deviation, there were no significant differences among the groups (*p* > 0.05).

**Figure 3:**
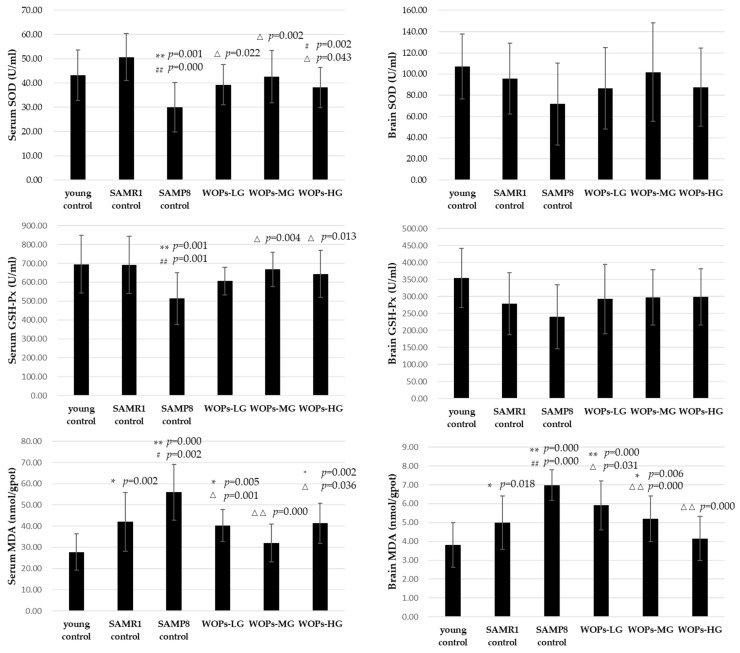
Effects of WOPs on serum SOD, GSH-Px and MDA, and brain SOD, GSH-Px and MDA. Data are presented as the means ± SD (*n* = 12). * *p* < 0.05 vs. the young control; ** *p* < 0.01 vs. the young control; ^#^ *p* < 0.05 vs. the SAMR1 control; ^##^ *p* < 0.01 vs. the SAMR1 control; ^Δ^ *p* < 0.05, ^ΔΔ^ *p* < 0.001 vs. the SAMP8 control.

Otherwise, compared to the mice in the young and SAMR1 control groups, the MDA content of serum and hippocampus tissue was markedly higher in the SAMP8 control group (*p* < 0.01). Compared with the SAMP8 control group, the MDA content of the serum was lower in WOPs-MG and WOPs-HG (*p* < 0.01 for WOPs-MG; *p* < 0.05 for WOPs-LG and WOPs-HG), while the MDA content of hippocampus tissue was greatly decreased in all WOP groups (*p* < 0.05) (shown in Figure 3).

### 3.4. Effects of WOPs on the Cholinergic Nervous System and Proinflammatory Factors in the Hippocampus Tissue

**Figure 4:**
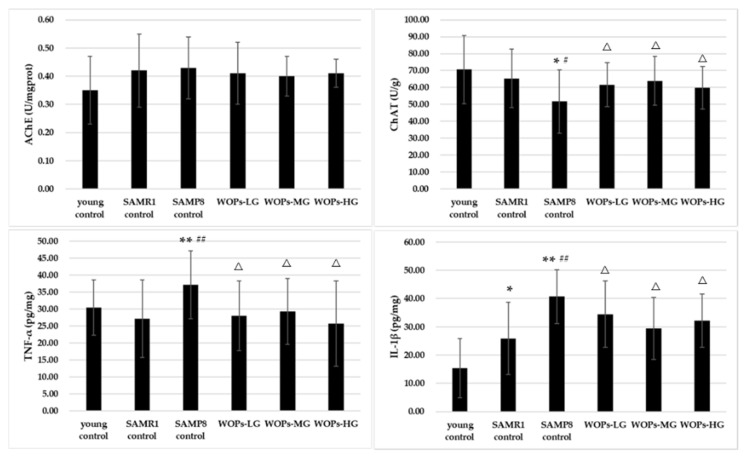
Effects of WOPs on hippocampus AChE, ChAT, TNF-α and IL-1β. Data are presented as the means ± SD (*n* = 12). * *p* < 0.05 vs. the young control; ** *p* < 0.01 vs. the young control; ^#^ *p* < 0.05, ^##^ *p* < 0.001 vs. the SAMR1 control; ^Δ^ *p* < 0.05 vs. the SAMP8 control.

A marked difference was found in ChAT activity among the three controls (*p* < 0.05); but no difference in AChE (shown in Figure 4). The treatment of WOPs significantly increased the ChAT activity of aging mice (*p* < 0.05). However, the AChE activity showed no difference among the WOP groups (*p* > 0.05). Compared with the young and SAMR1 controls, the concentrations of IL-1β and TNF-α were significantly increased in the SAMP8 control group (*p* < 0.01). Additionally, both IL-1β and TNF-α present a lower level in all the WOPs in comparison with SAMP8 control (*p* < 0.05).

### 3.5. Effects of WOPs on Phosphorylate Synaptic Plasticity, Neurotrophic Factors, and Aβ Generation in the Hippocampus Tissue

**Figure 5:**
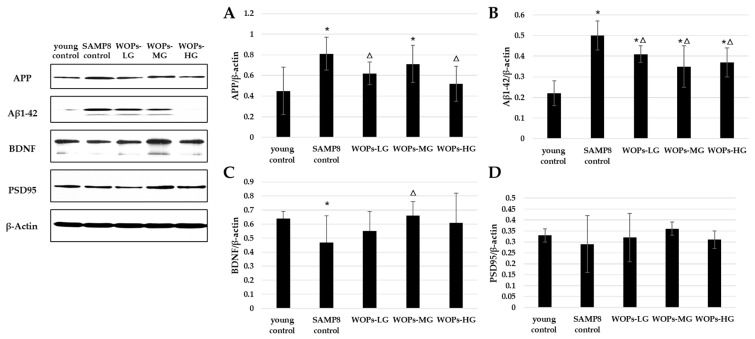
Effects of WOPs on the expression of APP, Aβ1-42, BDNF, and PSD95 in the hippocampus by Western blot; data were analyzed using a non-parametric test. Data are presented as the mean ± SD (*n* = 3 per group). * *p* < 0.05 vs. the young control; ^Δ^ *p* < 0.05 vs. the SAMP8 control.

To elucidate Aβ generation with aging, we evaluated the protein levels of APP and Aβ1-42 (shown in Figure 5A,B). With the aging process, the level of APP and Aβ1-42 was increased. Similarly, the expression of APP was significantly lower than that in the SAMP8 control group (*p* < 0.05). The expression of neurotrophic factors and synaptic proteins were detected in SAMP8 groups (shown in Figure 5C,D). Consistent with the behavioral results, the expression of BDNF and PSD95 was improved in WOP-treated groups in the comparison with SAMP8 age control (WOPs-MG vs. SAMP8 control, *p* < 0.05).

### 3.6. Effects of WOPs on Mitochondrial Function Factors and mtDNA Copy Number in the Hippocampus Tissue

**Figure 6:**
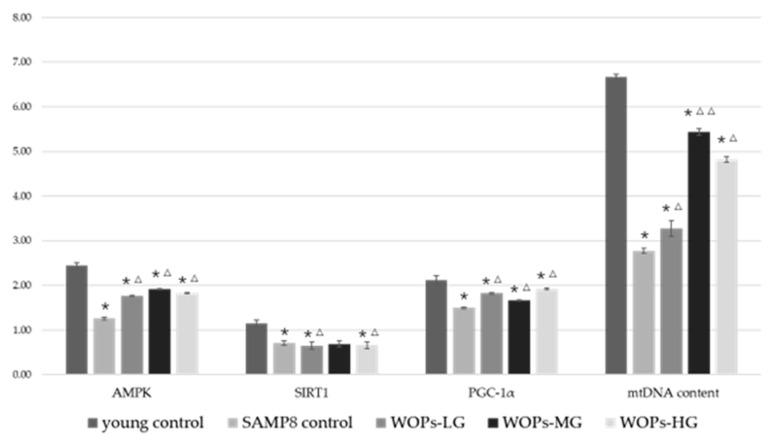
Effects of WOPs on mitochondrial function factors and relative mtDNA content in the hippocampus by real-time PCR analysis; data were analyzed using a non-parametric test. Data are presented as the mean ± SD (*n* = 5–6 per group). * *p* < 0.05 vs. the young control; ^Δ^ *p* < 0.05, ^ΔΔ^ *p* < 0.001 vs. the SAMP8 control.

As shown in Figure 6, mRNA expression of all mitochondrial biogenesis factors (AMPK, SIRT1 and PGC-1α) in aged mice was markedly lower than those in young control (*p* < 0.05). Moreover, mRNA expression of all mitochondrial biogenesis factors was increased in WOP groups, compared with the SAMP8 group (*p* < 0.05). The mtDNA content was also significantly improved in WOP groups after the WOPs treatment (*p* < 0.05). In particular, relative mtDNA content in WOPs-MG was 1.8-fold higher than in SAMP8 control (*p* < 0.01).

## 4. Discussion

Excessive accumulation of free radicals and mitochondrial dysfunction are typical features of brain aging and are considered good targets for early intervention. Walnuts are nuts with a variety of health benefits that are consumed worldwide [25,26,27,28]. Among these, the nootropic effect of walnuts is widely recognized [29,30,31]. In the past, most studies attributed the benefits of walnuts to walnut oil, with little focus on WOPs. This study shows that WOPs significantly improved aging-related decline in learning and memory.

In the present study, our behavioral experiments showed that SAMP8 mice showed a different degree of motor, exploration, learning, and memory impairment in the open-field test, the shuttle box test, the step-down test, and the Morris water maze test. In the open-field test, aging mouse models showed a decrease in squares crossed and reared. However, studies have shown that medium and high doses of WOPs considerably improved the number of squares crossed and standing time and improved age-related spatial exploration dysfunction in mice in open-field experiments. In the place navigation test, swimming speed decreased and escape latency did not significantly improve after six days of training. In the spatial probe test, escape latency was greatly extended in each WOP dose group, suggesting that WOPs have the potential to enhance learning and memory ability. In space exploration experiments, high-dose WOP intervention markedly improved the prolonged escape latency in SAMP8 mice and significantly increased the time spent in the target quadrant and the distance travelled in the target quadrant during the experiment. Although the escape response in all groups increased with time, indicating a continuous enhancement of learning, the number of times of active avoidance and electric shock increased in all aged mice in comparison with the young controls. In addition, these parameters decreased in all WOP-treated groups, suggesting that WOPs can enhance the weakened escape from electrical stimulation in old SAMP8 mice—the ability to improve conditioned reflexes. In the platform-jumping experiment, compared with the young group, there was no statistical difference in the number of mistakes in the SAMR1 control group. However, the number of errors in elderly SAMP8 mice increased significantly, and the latency time to stay on the platform was relatively short. WOPs-MG and WOPs-HG reduced the number of times that elderly mice received electric shocks, while the residence latency of elderly mice in the three WOP-intervention groups was ex-tended, reaching the level of the SAMR1 group. This indicates that WOPs can improve the reduction in passive avoidance responses in SAMP8 mice. Taken together, the results of our behavioral experiments suggest that WOPs improved age-related learning and memory disorders.

The maintenance of learning and memory function depends on the stability of the environment in hippocampus tissue. However, as hallmarks of aging, oxidative stress, chronic inflammatory and imbalance of mitochondrial homeostasis, no matter whether original occurring at systemic or central nervous system, are all potential mechanisms leading to brain function decline and presenting abnormal behavior. Several studies have shown that the increased oxidative stress in the aging brain is closely related to the decline of cognitive function, accompanied with a decrease in activities of SOD and GSH-Px, as well as an increase in MDA levels. In this study, compared with young and SAMR1 controls, the levels of antioxidant enzymes were decreased in the serum and the hippocampus of aged SAMP8 mice. WOPs-MG significantly enhanced the activity of serum SOD, and serum and hippocampus GSH-Px compared to SAMP8 control. These results indicate that WOPs improved the oxidative stress status of aging mice in both the serum and the hippocampus. These results indicate that WOPs effectively reduced oxidative damage to the hippocampus tissue by enhancing the antioxidant pathway, thereby maintaining hippocampus function. A chronic inflammatory state is one among the characteristics of aging and a risk factor for the high incidence of assorted age-dependent chronic diseases. Similarly, neuroinflammation of the central nervous system is an important reason for age-related learning and memory impairments. Studies have shown that there is a low level of immune activation in aging hippocampus tissue, expression of inflammatory factors were chronically elevated [32,33]. TNF-α is a vital pro-inflammatory cytokine. Additionally, the elevated levels of TNF-α within the hippocampus tissue would cut back the survival rate of newborn hippocampal neurons, result in impaired neurogenesis, and promote necrobiosis. In the present study, compared with the control group, there is abnormally high expression of the inflammatory factors IL-1β and TNF-α in the hippocampus and cortex of SAMP8 mice. However, the concentration of IL-1β in the hippocampus tissue of aged SAMP8 mice in the middle- and high-dose groups of WOP intervention was significantly decreased. Additionally, high-dose WOPs also significantly improved abnormally elevated TNF-α levels. Taken along the results of behavioral experiments, it may be speculated that WOPs could cut back the overexpression of age-related inflammatory factors and therefor the chronic inflammatory state of hippocampus tissue in aging people, thus reducing the incidence of inflammation-dependent hippocampal dysfunction.

As a universally recognized biomarker of Alzheimer’s disease, Aβ was deposited in senile plaques as an amorphous aggregate of amyloid fibrillary or non-fibrillary, thus forming the typical brain features of Alzheimer’s disease [34]. Aβ is produced by hydrolysis of amyloid precursor protein APP in vivo, mainly including Aβ1-42, Aβ1-40 and Aβ1-43. Among them, Aβ1-42 is the main type of Aβ and also the main component of the senile plaques in the brain of AD patients [35,36]. The neurotoxicity of Aβ is a common pathway of multiple factors leading to the pathogenesis of AD, which can cause a series of intracellular physiological and biochemical changes [37,38,39]. Aβ can induce the generation of oxygen-free radicals, and oxygen-free radicals can also promote the decomposition of APP into Aβ. Aβ can prolong cell depolarization by inhibiting potassium channels and cause voltage-dependent calcium channel opening, leading to intracellular calcium overload. Meanwhile, Aβ resulted in the permeability of mitochondrial membrane potential and PTP (permeability transition pore) formation. In recent years, the focus of Aβ research has gradually shifted from insoluble amyloid plaques to soluble oligomers. Studies have found that Aβ oligomer is the main cause of neurotoxicity, resulting in the decline of learning and memory in AD patients. In the early stages of AD, large amounts of Aβ oligomers induce a series of biochemical changes, leading to a decrease in the synaptic plasticity of nerve cells and the ability to learn and memory. SAMP8 mice produced excessive soluble Aβ spontaneously in the hippocampus after the growth period, accompanied by a rapid decline in learning and memory. The study showed that, similar to AD, it was these soluble Aβ that caused the decline in learning and memory in SAMP8 mice. The expression levels of APP protein in the WOP group were significantly lower than that in the SAMP8 control group. The expression of Aβ1-42 protein in WOP groups was significantly lower than that in SAMP8 control groups. These results suggest that WOPs can interfere with the potential damage to hippocampal neurons and synaptic plasticity by reducing amyloid deposition in brain tissue, which has the potential to slow the development of age-dependent learning and memory impairment and reduce the risk of neurodegenerative diseases. BDNF is a very important member of the nerve growth factors (NGFs) family. It is widely found in the nervous system, especially in the hippocampus. The level of BDNF is decreased with an increase in age, and the persistent inflammatory response common in the elderly can reduce the expression of BDNF, which makes it a key mechanism to regulate age-related neural dysfunction. In this study, the expression level of BDNF in WOPs-MG groups was significantly higher than that in non-nucleotide and ordinary control groups, indicating the protective effect of WOPs. Postsynaptic density protein (PSD) is a specialized region composed of multiple proteins located under the postsynaptic membrane of the central nervous system. Under the electron microscope, PSD shows an increased density shadow and plays an important role in mediating and integrating synaptic signal transmission and learning and memory. PSD95 is a special intracellular protein in PSD and the main framework component of PSD. The expression level of PSD95 in the SAMP8 control group was lower than that in other groups, but there was no significant difference.

Mitochondria are among the foremost necessary organelles in neurons, and turn out ATP to support their physiological activity. Varied studies have disclosed alterations of mitochondria relating to aging, including mitochondrial enlargement, depolarization of membrane potential, and decreased ATP production [40,41,42]. Nerves require continuous ATP production to maintain long-term neural activity and electrical conduction. Mitochondria not only seem to be the most site of oxidative phosphorylation and ATP production but also play a vital regulative role in oxidative stress [43]. Mitochondrial dysfunction in the nervous system has been linked to neurodegenerative diseases. PGC-1α is a transcriptional co-activator concerned in mitochondrial biogenesis, OXPHOS, antioxidant defense, and different processes and is closely associated with the incidence and development of neurodegeneration [44]. Researchers have found that SIRT1 is neuroprotective in AD models by regulating Aβ metabolism, and its deletion causes increased tau acetylation and phosphorylation, and cognitive defects [45]. In mitochondria, Biogenesis and electron transport systems could be regulated by upregulation of the expression of SIRT1 and PGC-1α [46]. Similarly, the present study showed a positive association between WOP supplementation and upregulation of SIRT1 and PGC-1. As an upstream target of a series of phosphorylation-dependent adaptive modifiers, AMPK works together with PGC-1α to activate catabolic pathways to produce ATP and suppress energy consumption. It has been reported that cognitive ability could be improved by regulating neuronal mitochondrial homeostasis including mitophagy and mitochondrial biogenesis [47,48,49]. Likewise, an interesting finding of the present study was that AMPK expression was upregulated in the hippocampus tissue of mice after WOP treatment. These results imply a neuroprotective role of WOPs by promoting mitochondrial biogenesis via the AMPK/SIRT1/PGC-1 signaling pathway. mtDNA copy number is taken into account as a surrogate marker of mitochondrial function [50]. In this study, we found that WOPs improved mitochondrial function in the hippocampus by restoring mtDNA content and increasing AMPK/SIRT1/PGC-1α expression.

Abnormal levels of AChE and ChAT in the hippocampus of AD patients cause cholinergic neuron loss and degeneration of cholinergic fibers [51]. ChAT is a promoter of cholinergic function and promotes the synthesis of acetylcholine from acetyl-CoA and choline. In this study, hippocampus AChE activity was considerably augmented and hippocampus ChAT activity was considerably decreased in SAMP8 aged mice with learning and memory dysfunction, suggesting the occurrence of cholinergic dysfunction. WOPs significantly increased ChAT levels. However, there was no significant difference in AChE activity between the WOP-treated and aging control groups. Thus, further research is needed to determine the effects of WOP supplementation on age-dependent acetylcholine-related disorders.

Our study had many limitations. First, the dynamic intervention impact continues to be unclear, because these are the results of just one sample. Second, there was no examination of mitochondrial morphology, since we did not perform microscopy. Third, though the results of this study determined, in vivo, the efficiency and dose effect of WOPs on learning and memory, dose determination in humans is still unclear. Therefore, further clinical trials and primary cell culture experiments should be performed to confirm the observed effects, for a deeper investigation of the mechanism, and for application in patients.

## 5. Conclusions

Our findings strongly indicated a significant beneficial effect of WOPs on age-related learning/memory dysfunction. We speculate that the effect of WOPs could also be associated with improved mitochondrial function via the AMPK/SIRT1/PGC-1α pathway, therefore reducing the expression of inflammatory factors and anti-oxidative injury in the aging hippocampus. Clinical trials should be performed to verify WOP efficacy and optimal dose in humans.

## Figures and Tables

**Table 1:** Effects of WOPs on the space exploration capability.

Groups	No. of Rearing	No. of Squares Crossed	No. Fecal Boli	No. of Inner Squares Entered	Time Spent in Inner Squares (s)
young control	5.1 ± 8.4	61.9 ± 38.8	1.8 ± 1.7	3.1 ± 3.1	12.82 ± 15.00
SAMR1 control	4.1 ± 2.0	92.9 ± 25.9	1.8 ± 1.4	1.1 ± 1.0	2.35 ± 3.50
SAMP8 control	4.3 ± 5.6	71.8 ± 49.8	1.0 ± 1.3	1.2 ± 1.5	6.59 ± 8.50
WOPs-LG	1.8 ± 2.1	46.3 ± 36.9 ^#^	1.4 ± 1.2	1.1 ± 1.0	8.16 ± 9.09
WOPs-MG	2.3 ± 4.1	36.9 ± 33.2 ^##^	2.1 ± 1.8	0.8 ± 1.0	5.77 ± 9.99
WOPs-HG	1.2 ± 1.5 *	26.8 ± 18.3 *^##^	2.3 ± 2.1	1.1 ± 1.2	5.50 ± 8.65

Data are presented as the means ± SD (*n* = 12). * *p* < 0.05 vs. the young control; ^#^ *p* < 0.05, ^##^ *p* < 0.001 vs. the SAMR1 control.

**Table 2:** Effects of WOPs on the learning and memory capability of the place navigation test.

Groups	Time Spent in the Target Quadrant (s)	Times of Platform Crossed
young control	18.30 ± 4.14	3.4 ± 1.5
SAMR1 control	16.23 ± 6.30	1.8 ± 1.5 *
SAMP8 control	6.61 ± 6.54 *^#^	0.9 ± 1.6 **
WOPs-LG	13.69 ± 8.20 ^Δ^	0.3 ± 0.7 **^#^
WOPs-MG	20.12 ± 9.28 ^ΔΔ^	0.8 ± 1.4 **
WOPs-HG	15.30 ± 7.93 ^Δ^	2.3 ± 1.9 ^Δ^

Data are presented as the means ± SD (*n* = 12). * *p* < 0.05, ** *p* < 0.001 vs. the young control; ^#^ *p* < 0.05l; ^Δ^ *p* < 0.05, ^ΔΔ^ *p* < 0.001 vs. the SAMP8 control.

**Table 3:** Effects of WOPs on the number of errors and latency.

Groups	the Number of Errors (No.)	Latency (s)
young control	1.2 ± 0.4	206.08 ± 63.98
SAMR1 control	1.4 ± 0.5	171.63 ± 54.35
SAMP8 control	3.1 ± 1.2 *^#^	71.49 ± 55.60 *^##^
WOPs-LG	1.8 ± 1.0	147.46 ± 55.50 *^Δ^
WOPs-MG	1.5 ± 0.7 ^Δ^	165.85 ± 45.54 ^ΔΔ^
WOPs-HG	1.6 ± 0.7 ^Δ^	163.34 ± 45.69 ^ΔΔ^

Data are presented as the means ± SD (*n* = 12). * *p* < 0.05 vs. the young control; ^#^ *p* < 0.05, ^##^ *p* < 0.001 vs. the SAMR1 control; ^Δ^ *p* < 0.05, ^ΔΔ^ *p* < 0.001 vs. the SAMP8 control.

## Data Availability

The data presented in this study are available on request from the corresponding author. The data are not publicly available due to privacy. The studies not involving humans.
